# An updated report on the incidence and epidemiological trends of keratinocyte cancers in the United Kingdom 2013–2018

**DOI:** 10.1002/ski2.61

**Published:** 2021-08-18

**Authors:** M. Kwiatkowska, S. Ahmed, M. Ardern‐Jones, L. A. Bhatti, T. O. Bleiker, A. Gavin, S. Hussain, D. W. Huws, L. Irvine, S. M. Langan, G. W. M. Millington, H. Mitchell, R. Murphy, L. Paley, C. M. Proby, C. S. Thomson, R. Thomas, C. Turner, S. Vernon, Z. C. Venables

**Affiliations:** ^1^ National Cancer Registration and Analysis Service London UK; ^2^ British Association of Dermatologists London UK; ^3^ Department of Clinical Experimental Sciences Faculty of Medicine University of Southampton Southampton UK; ^4^ Public Health Scotland Edinburgh UK; ^5^ University Hospitals of Derby and Burton NHS Foundation Trust Derby UK; ^6^ North Ireland Cancer Registry Belfast UK; ^7^ Public Health Wales Cardiff UK; ^8^ London School of Hygiene & Tropical Medicine St. John's Institute of Dermatology London UK; ^9^ Department of Dermatology Norfolk and Norwich University Hospital Norwich UK; ^10^ University of East Anglia Norwich Medical School Norwich UK; ^11^ Sheffield Teaching Hospitals NHS Foundation Trust Sheffield UK; ^12^ Department of Dermatology Ninewells Hospital and Medical School Dundee UK

## Abstract

**Introduction:**

The most common cancers in the UK are keratinocyte cancers (KCs): the combined term for basal cell carcinomas (BCCs) and cutaneous squamous cell carcinomas (cSCCs). Registration of KC is challenging due to high numbers and multiplicity of tumours per person.

**Methods:**

We provide an updated report on the descriptive epidemiology of trends in KC incidence for the resident populations of UK countries (England, Northern Ireland, Scotland and Wales) using population‐based cancer registry and pathology report data, 2013–18.

**Results:**

Substantial increases in cSCC incidence in England, Scotland and Northern Ireland can be detected for the period of 2013–18, and the incidence of cSCC also increased in Wales from 2016 to 2018. In contrast, however, the pattern of annual change in the incidence of BCC across the nations differs. In England, the incidence of BCC declined slightly from 2016 to 2018, however, the overall trend across 2013–18 is not statistically significant. In Scotland, the incidence of BCC shows some variability, declining in 2017 before increasing in 2018, and the overall trend across 2013–18 was also not statistically significant. In Northern Ireland, the incidence of BCC increased significantly over the study period, and in Wales, the incidence of BCC increased from 2016 to 2018. One in five people will develop non‐melanoma skin cancers (NMSC) in their lifetime in England. This estimate is much higher than the lifetime risk of melanoma (1 in 36 males and 1 in 47 females born after 1960 in the UK), which further highlights the burden of the disease and importance of early prevention strategies.

**Conclusions:**

We highlight how common these tumours are by publishing the first ever lifetime incidence of NMSC. Additionally, the first time reporting of the age standardised incidence of KC in Wales further confirms the scale of the disease burden posed by these cancers in the UK. With approximately one in five people developing NMSC in their lifetime, optimisation of skin cancer prevention, management and research are essential.

## Graphical abstract

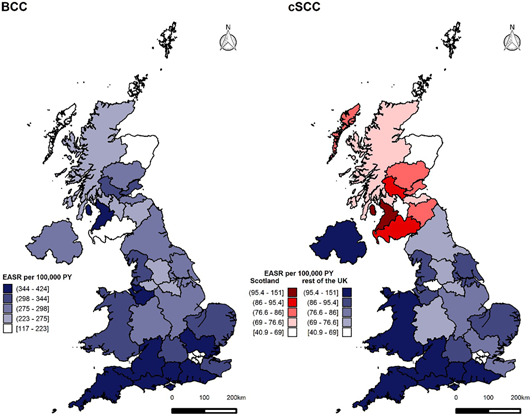

The commonest skin cancers are keratinocyte cancers (KCs), the combined term for basal cell carcinomas (BCC) and cutaneous squamous cell carcinomas (cSCC). We provide an updated report on KC incidence and epidemiological trends in the United Kingdom using data from national cancer registries in England, Scotland, Northern Ireland and Wales in 2013‐18. We highlight how common these tumours are by publishing the first ever lifetime incidence of NMSC, which indicates that one in five people in England will develop a NMSC at some point in their lifetime.

1

**What’s already known about this topic?**

- Keratinocyte cancers (KCs) are the commonest cancers in white populations, with rates increasing worldwide.
- These tumours are regularly excluded from official national cancer statistics.
- Improved data collection methods and validated tumour reporting techniques facilitated reporting of a more accurate incidence of KC in the United Kingdom (UK) (excluding Wales) in 2013–16.

**What does this study add?**

- We provide an updated report on KC incidence and epidemiological trends in the UK using data from national cancer registries in 2013–18.
- The incidence of cutaneous squamous cell carcinoma continues to raise in all UK countries, however the annual change in incidence of basal cell carcinoma differs by nations.
- We highlight how common these tumours are by publishing the first ever lifetime incidence of non‐melanoma skin cancers (NMSC), which indicates that one in five people in England will develop a NMSC at some point in their lifetime.
- Additionally, the first ever reporting of the incidence of KC in Wales further confirms the scale of the disease burden posed by these cancers on the UK nations.

## 2 INTRODUCTION

Skin cancers are the most common type of cancer in the United Kingdom (UK).^1^ Keratinocyte cancers (KC) are a group of non‐melanoma skin cancers (NMSC) that include basal cell carcinomas (BCC) and cutaneous squamous cell carcinomas (cSCC). Other NMSC include rarer skin cancers such as Merkel cell carcinoma. KC are 10 times more common than melanoma and make up a large proportion of dermatology workload. In England, dermatologists see more urgent ‘two‐week wait’ cancer referrals than any other speciality.^2^ Therefore, accurate data on KC incidence is crucial for adequate dermatology service planning, and to estimate the population burden of disease and the potential for prevention in the UK.

Three factors contribute to the previous lack of good epidemiological data regarding KC:

1. Perceived low priority linked with excellent survival compared to melanoma;
2. High frequency of tumours resulting in very high registration workload;
3. Complexity of registering multiple tumours per patient accurately and lack of access to easily extractable structured pathology data.

As a result, KC incidence are often excluded from cancer epidemiology reporting, research and in planning of public health interventions. Although BCC net survival is sometimes shown to be 100% or higher, cSCC survival may be more comparable to that of low risk melanoma.^3^ Net survival greater than 100% indicates that patients in this group have a better chance of surviving the defined number of years after diagnosis compared with that expected for their age and sex and is over 100% in other cancers such as stage I prostate cancer. For the reasons above, the number of cases of BCC and cSCC registered is an underestimation of the true tumour incidence because of the current United Kingdom and Ireland Association of Cancer Registries rule recommending that only the first occurrence of BCC and/or cSCC in an individual be registered (1st tumour per patient all‐time [1st PP]). This is comparable to most national cancer registry rules.^4^ A notable exception to this rule is the registration of cSCC in Scotland where all instances of these tumours are manually registered.

We previously linked available population cancer registry data for the first tumour record for BCC and cSCC to subsequent BCC and cSCC pathology reports and reported the 1st BCC and cSCC per patient per annum (1st PPPA) to better represent the true tumour incidence as proposed by Venables et al.^5^ The 1st PPPA method relies on the registry linkage to subsequent BCC and cSCC pathology reports, which are linked to the first tumour record. The technique better represents the population tumour incidence than 1st PP method. Using the Public Health England (PHE) National Cancer Registration and Analysis Service (NCRAS) data, we reported 50% more tumours than the 1st tumour per patient all‐time method, estimated to be within the 10% of the true tumour incidence, without additional cancer registration workload.

Skin cancer data collection has improved in England due to introduction of the auto‐processing of BCC and cSCC and nationalisation of cancer registration in 2013. The system automatically creates cancer registration records for BCC and cSCC following the extraction of data from full‐text pathology records and Cancer Outcomes and Services Dataset (COSD) records, which minimises the burden of manual processing. The improved data collection and linkage to pathology reports enables more accurate estimation of KC incidence as an alternative to manually registering all tumours which would require resources that are not routinely available to the registry. A similar system to that in England has recently been introduced in Wales by the Welsh Cancer Intelligence and Surveillance Unit (WCISU) of Public Health Wales. This has resulted in significantly improved and complete population skin cancer registration data in Wales from 2016 incidence onwards.

This first ever report of the population incidence of KC in the United Kingdom between 2013 and 18 has been possible using patient data processed by all the UK cancer registries led by a British Association of Dermatologists (BAD) funded analyst in partnership with NCRAS, PHE and in collaboration with analysts and directors of the other UK cancer registries. Our aims are to improve skin cancer registration and the reporting of reliable epidemiological data to aid research, the provision of dermatological services and public health interventions. Analysed aggregated data presented in this report are available in the Supplementary Material.

## 3 MATERIALS AND METHODS

### 3.1 Study design, setting and participants

It is a descriptive epidemiology study of trends in KC incidence rates for resident population of UK countries England, Northern Ireland, Scotland and Wales using population‐based cancer registry and pathology report data.

English population‐based cancer registry data were provided by NCRAS, England.^6^ All NHS pathology laboratories are required to submit cancer pathology reports to NCRAS. Laboratory compliance is monitored by the NCRAS Data Quality Team. Pathology reports in combination with information from Patient Administration System (PAS) and Cancer Outcomes and Services Dataset (COSD) form a new cancer registration record. The auto‐processor in England registers around 75% BCC and cSCC and all remaining tumours are registered manually.

Population‐based cancer registry data for Scotland was provided by Public Health Scotland (PHS) (BCC 1st PPPA incidence, 2013–18 and publicly available BCC 1st all‐time data and all registered cSCC data 2013–18). Welsh population registry data was provided by the WCISU of Public Health Wales (KC 1st PPPA incidence, 2016–18, data for 2013–15 was not available due to incomplete data collection). The Northern Ireland Cancer Registry provided data for 2013–18 KC incidence using both 1st PP and 1st PPPA methods (Table 1).

**TABLE 1:** Keratinocyte cancer data including counting method and time period provided by cancer registries in the UK

Method	Country			
	England	Scotland	Wales	Northern Ireland
1st all‐time	BCC 2013–18 (look back period started in 1995)	BCC 2013–18 (look back period started in 1960, but more complete data from 1975)	X	BCC 2013‐18 (look back period started in 1993)
	cSCC 2013–18 (look back period started in 1995)	X	X	cSCC 2013–18 (look back period started in 1993)
1st per patient per annum	BCC 2013–18	BCC 2013–18	BCC 2016–18	BCC 2013–18
	cSCC 2013–18	X	cSCC 2016–18	cSCC 2013–18
All registered	X	X	X	X
	X	cSCC 2013–18	X	X

Abbreviations: BCC, basal cell carcinomas; cSCC, cutaneous squamous cell carcinomas.

### 3.2 Variables

BCC and cSCC tumours were identified by ICD‐10 and ICD‐O2 morphology codes and standard inclusion criteria (Appendix 1: Cohort definition). The KC incidence was calculated using three methodologies: the first per patient all‐time (1st PP), first per patient per annum (1st PPPA) method and all‐registered tumours. Briefly, 1st all‐time method ranks BCC and cSCC tumours per patient in chronological order going as far back in time as is available in the cancer registry data and selects the first tumour of that subtype (i.e., BCC or cSCC) received by the registry (See Table 1). 1st PPPA method ranks tumours per patient in chronological order within each calendar year and selects the first tumours of that subtype of each calendar year. The cohort was subsequently limited to the first tumours diagnosed in 2013–18 for all methods (in Wales, we were limited to 2016–18 due to incomplete data for years before 2016; therefore, we are restricted to 1st PPPA data due to insufficient look back period to determine first tumour per patient). Scottish incidence data was based on 1st PPPA incidence 2013–18 for BCC and publicly available data on PHS website for 1st PP incidence 2013–18 looking back to 1975 to determine the first tumour per patient. cSCC data in Scotland corresponds neither to 1st PP nor 1st PPPA counting methods, because all cSCC tumours and not just the first tumour per patient were registered in Scotland.

Date of diagnosis of the first BCC and cSCC per patient per annum (1st PPPA) tumours was determined as the date when sample was taken, received or reported by the laboratory (whichever was not missing and came first).

Patient demographics such as age, sex and self‐reported ethnicity were derived from National Cancer Registration Dataset in England.^7^ Deprivation quintiles for patients resident in England were calculated using the patient’s Lower Super Output Area at diagnosis and linked to the income domain scores of the Index of Multiple Deprivation 2019. The country was split into small areas, and an income score was assigned to each area. The areas were then grouped into quintiles according to their deprivation score, with approximately 20% of the total population in each quintile (rather than 20% of the areas in each quintile). The quintiles were ordered from least deprived (1) to most deprived (5).^7^ Ethnicity was self‐reported and coded using PAS embedded in NHS hospitals. Geographies were defined based on patients' postcode of residence at Cancer Alliance level in England and NHS Board in Scotland.

### 3.3 Statistical analysis

Incidence and European Age‐Standardised Rates (EASRs) were prepared from the four national cancer registries following the established methodology. Data for England and Wales were extracted using Oracle SQL developer.

Directly age and sex standardised rates were computed using the European Standard Population 2013 and expressed per 100 000 person‐years at risk. EASR was calculated using ESP2013 and using 5‐year age groups 0–4, 5–9 up to an upper age group of 90+. For each age band of each population being compared, age‐specific incidence rates were multiplied by the size of the standard population for that age band. EASRs were calculated by dividing the total number of expected cases by the total standard population size. Confidence intervals for EASRs have been calculated according to the Binomial distribution using a formula which works only when numbers are sufficiently large, that is, more than 20 cases.

A statistical test for trend was performed for English, Scottish and Northern Irish data to test whether the EASR incidence for BCC and cSCC was changing over time. The statistical tests were performed in R statistical software using Poisson and robust linear regression using glm (generalised linear model) function from stats package and robust linear model (rlm) function from MASS package.

Other statistical tests were performed on English data only, using 1st all‐time KC count. The results were deemed significant at 5% significance level. Significance level was adjusted to account for multiple testing where necessary. We aimed to test whether:

- BCC and cSCC are more common in males than females.
- BCC and cSCC are more common in females than males under the age of 50.
- Incidence of BCC and cSCC in people under the age of 50 is increasing over time, and how this varies by sex.
- BCC and cSCC incidence increase with age.
- EASR for BCC and cSCC differs by deprivation level.

The results were shown as a linear regression coefficient *β* and Poisson regression coefficient transformed into an incidence rate ratio accompanied by 95% confidence interval.

Lifetime risk of KC in England was calculated using the method based on current probability developed at Cancer Research UK using a technique described by Sasieni et al using only the first all‐time NMSC tumour registered in 2013–18 in England.^8^, ^9^ Data are limited to NMSC analysis as mortality data is provided only for NMSC as a single category which may include rare NMSC such as Merkel cell carcinoma. The method uses a period approach to calculating lifetime risk that assumes constant incidence rates through each person’s lifetime. The calculation approximates the risk over a theoretical lifetime; it assumes that for the whole of the theoretical lifetime, the risk of skin cancer is the same as it was between 2013 and 18. If skin cancer rates had remained static, the lifetime risk calculated would model quite closely the lifetime risk of the average person. However, skin cancer risk has increased over time. While this calculation provides an indicative risk of skin cancer over a lifetime, this limitation of the period methodology should be understood, and it should not be interpreted as an actual lifetime risk for an individual person.

### 3.4 Validation

100 patients with BCC and 100 patients with cSCC in England in 2018 were randomly selected for review by a single dermatologist (Zoe C. Venables). Pathology reports of all tumours recorded that year for the patient were reviewed to determine the accuracy of registration and the counting methods.

Additionally, we compared tumour count of 1st PP and 1st PPPA methods and reported a percentage difference to aid the interpretation of the methods.

## 4 RESULTS

### 4.1 First per patient all‐time incidence

#### 4.1.1 Patient characteristics, England

In England, patients with 1st all‐time BCC and cSCC were more commonly males with male to female ratios of 1.2:1 and 1.7:1, respectively, using counts. The median age at time of first incidence was 71 years for BCC (IQR 62–80) and 79 years for cSCC (IQR 71–85).

The majority of patients were of white ethnicity with 19.2% of patients with unknown ethnicity and fewer than 1.0% patients self‐reported as non‐white. Both BCC and cSCC are more common in least deprived quintiles; 27.0% of patients with a first BCC diagnosis lived in the least deprived areas and 11.0% lived in the most deprived areas. Similarly, 25.8% of patients with cSCC lived in the least deprived areas and 11.5% lived in the most deprived areas.

### 4.2 For the years 2013–2018

**FIGURE 1:**
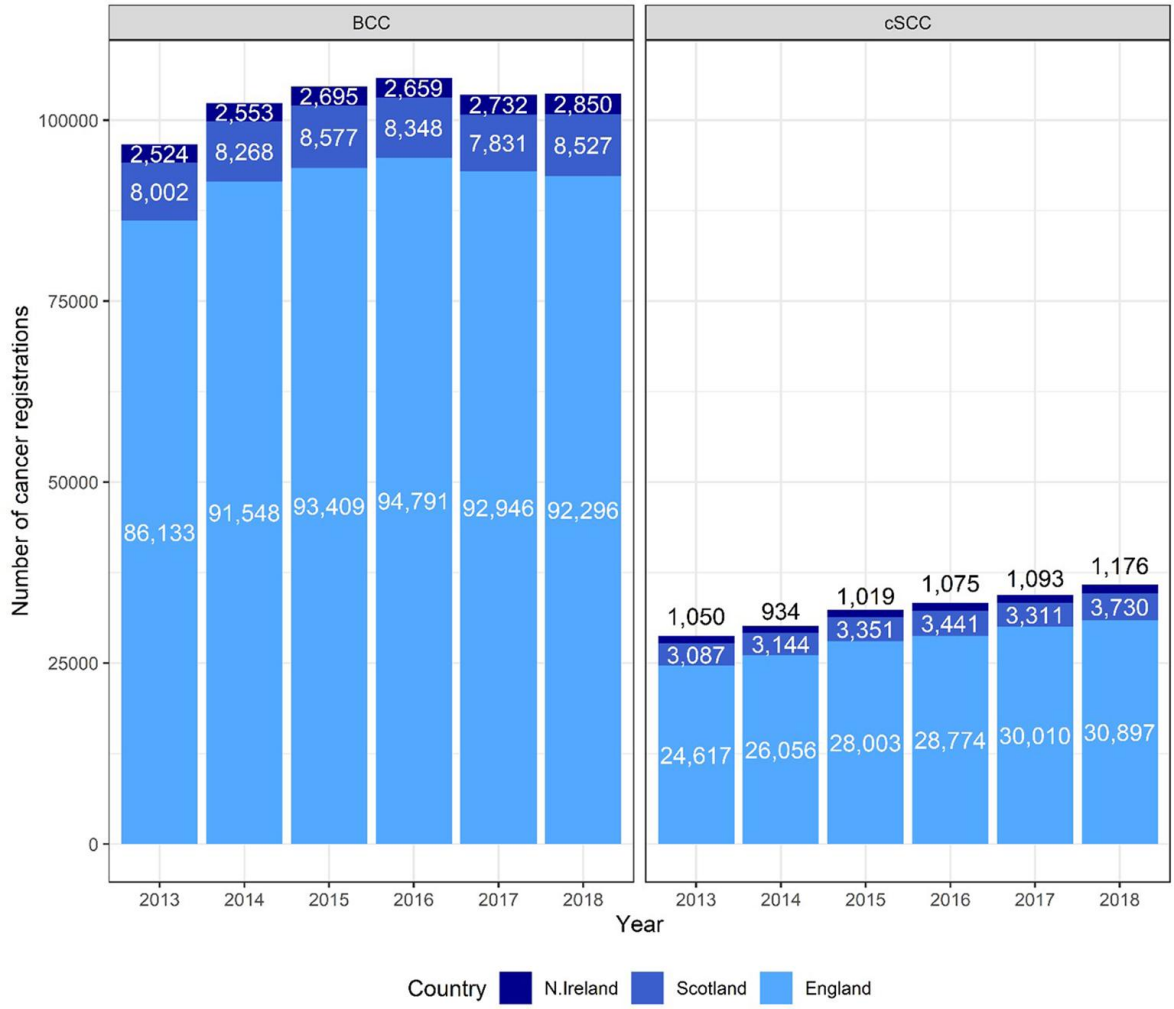
Basal cell carcinoma and cutaneous squamous cell carcinoma (cSCC) tumour count using the first per patient all‐time technique for England, N. Ireland and Scotland, except for cSCC count in Scotland, where all instances of cSCC are registered

Using first tumour registered per patient all‐time method, there were 91 854 diagnoses of BCCs per year on average and 28 060 cSCC per year on average in England (551 123 and 168 357 over the 6‐year period) (Figure 1). The average annual percentage change in tumour count for the period of 2013 to 2015 for BCC and cSCC was 4.2% and 6.7%, respectively. New data from 2016 to 2018 shows that the BCC tumour count dropped by 0.4% on average from 2016 to 2018, whereas the cSCC tumour count increased by 3.3% on average in the same period (See Supplementary Data File).

In England, the EASR of BCC was 215.7 per 100 000 person‐years (PY) in males (95% CI 214.9, 216.5) and 155.5 PY (95% CI 154.9, 156.1) in females. The EASR of cSCC was 81.9 PY (95% CI 81.4, 82.4) in males and 36.2 PY (95% CI 35.9, 36.5) in females.

The incidence rate of BCC decreased by an average of 1.2 PY, which was statistically non‐significant (95% CI ‐4.6, 2.3) while the incidence rate of cSCC increased by an average of 1.4 PY over the study period (95% CI 0.7, 2.2).

In Scotland, the average count of BCC using the 1st all‐time method was 8259 and the average count of cSCC was 3344 where all instances of cSCC are registered (Figure 1). The EASR of BCC was 192.8 PY (95% CI 190.4, 195.2) in males and 135.0 PY (95% CI 133.2, 136.7) in females. The EASR of cSCC was 112.0 PY (95% CI 110.1, 114.0) in males and 36.5 PY (95% CI 35.6, 37.4) in females. All instances of cSCC are registered in Scotland.

In Northern Ireland, the average count of BCC using the 1st all‐time method was 2669 and the average count of cSCC was 1058. The EASR of BCC was 211.1 PY (95% CI 206.6, 215.5) in males and 137.6 PY (95% CI 134.4, 140.8) in females. The EASR of cSCC was 106.8 PY (95% CI 103.4, 110.1) in males and 44.2 PY (95% CI 42.4, 46.0) in females.

Welsh data collection of BCC and cSCC is incomplete for years prior to 2016 and, therefore, it not possible to determine the first tumour per patient due to insufficient look back period. We are limited to 1st PPPA data in Wales.

### 4.3 First PPPA

Using the 1st per patient per annum method, there were 146 852 diagnoses of BCC per year on average and 39 017 cSCC per year on average in England in 2013 to 2018 (881 112 and 234 103 over the 6‐year period).

**FIGURE 2:**
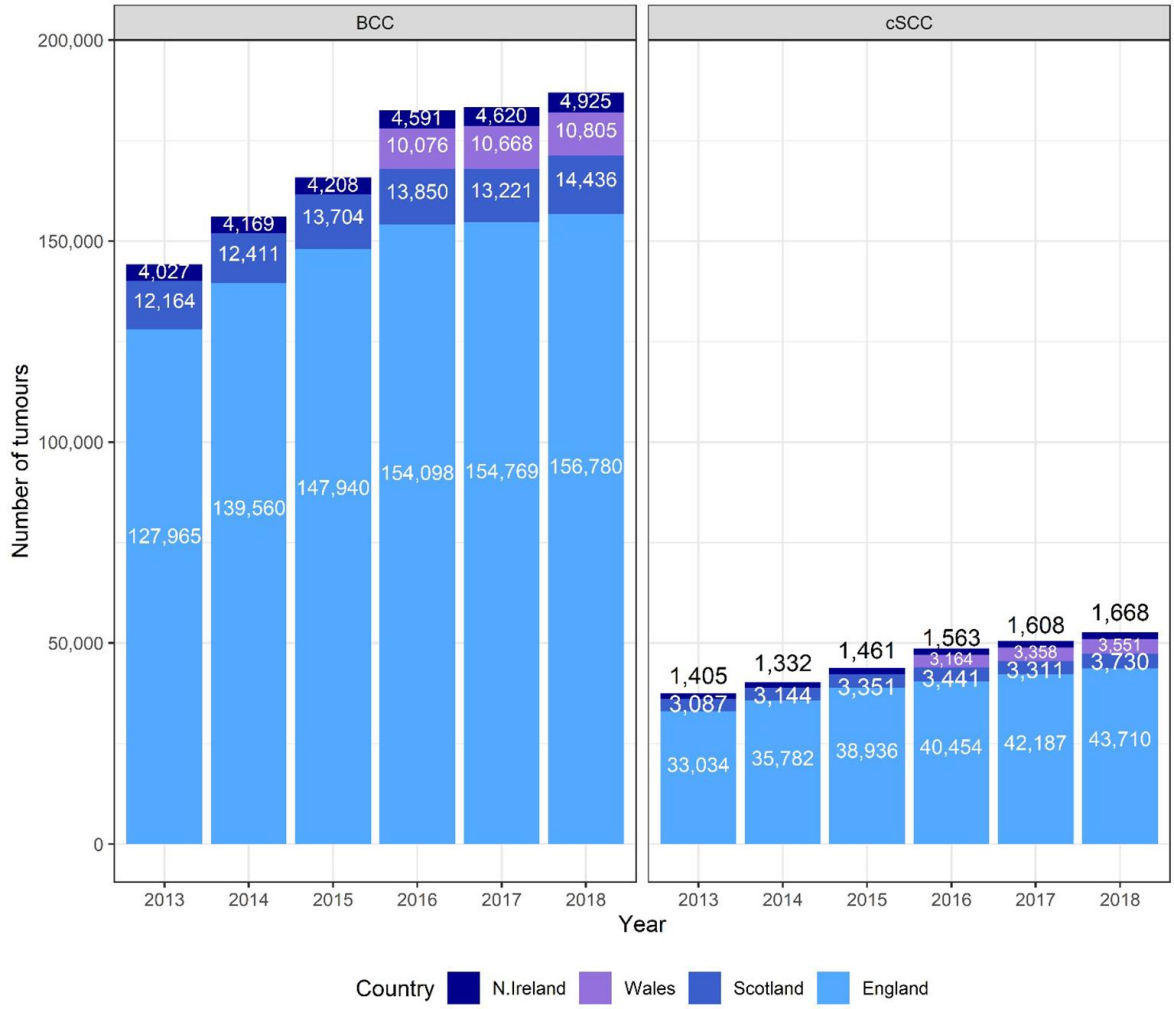
Basal cell carcinoma and cutaneous squamous cell carcinoma (cSCC) tumour count using first per patient per annum technique for England, Wales, N. Ireland and Scotland, except for cSCC count in Scotland, where all instances of cSCC are registered. Data for Wales available for 2016–18 only

In Scotland, the average count of BCC using the 1st PPPA method was 13 300 from 2013 to 2018 (79 786 over the 6‐year period) (Figure 2). All cSCC are manually registered in Scotland with 3344 on average per year from 2013‐2018 and 20 064 over the 6 year period.

In Northern Ireland, the average count of BCC and cSCC using the 1st PPPA method were 4423 and 1506 from 2013 to 2018 (26 540 and 9037 over the 6‐year period).

In Wales, the average count of BCC and cSCC using the 1^st^ PPPA method were 10 516 and 3358, respectively, from 2016 to 2018 (31 549 and 10 073 over the 3‐year period). Welsh data for previous years was not available.

**FIGURE 3:**
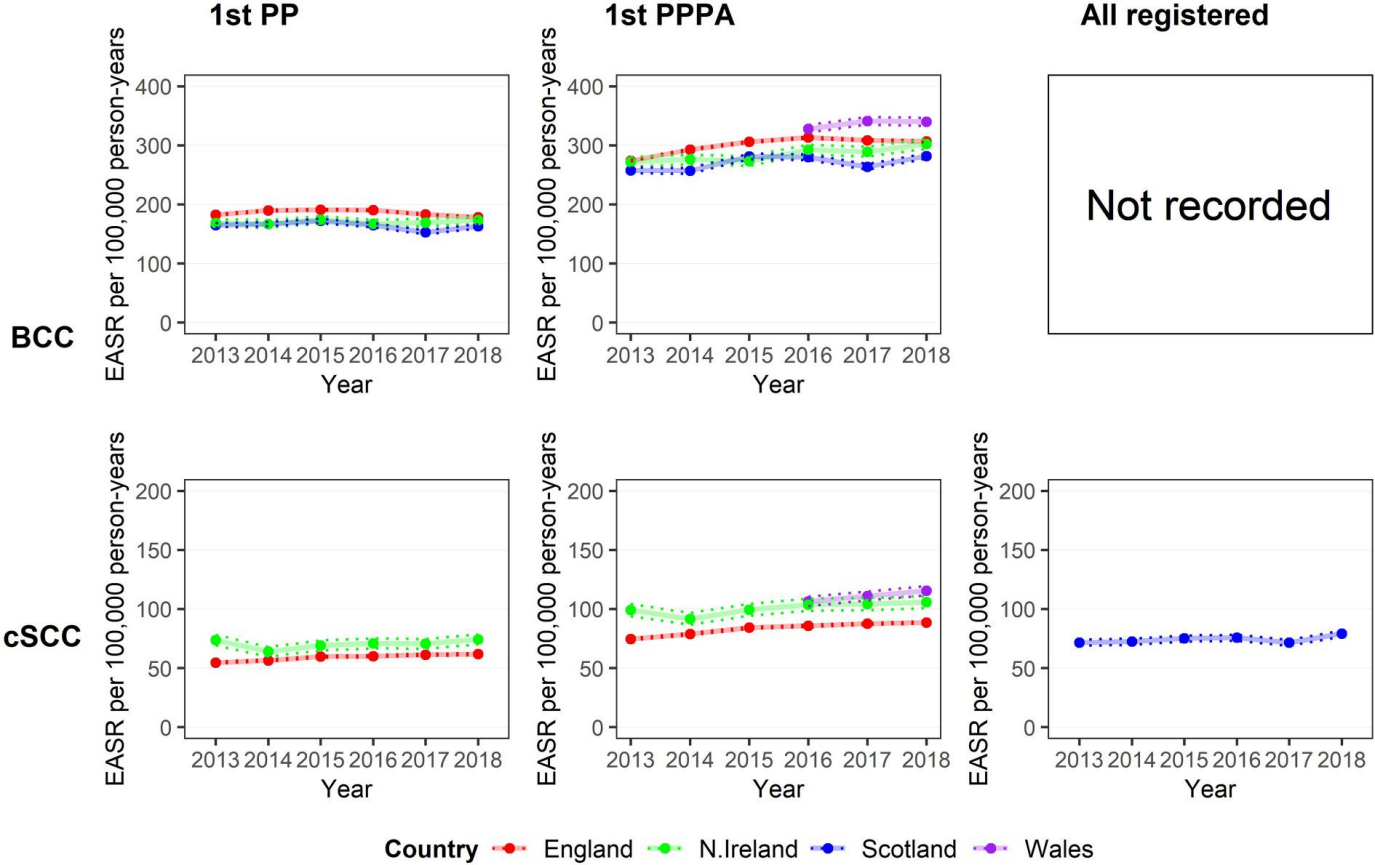
National incidence rate of basal cell carcinoma (BCC) and cutaneous squamous cell carcinoma (cSCC) based on three counting techniques. Column 1. National European Age‐Standardised Rate (EASR) of BCC (top) and cSCC (bottom) 2013–18, using 1st per patient all‐time (1st PP) technique. Column 2. National EASR of BCC and cSCC 2013–18, using 1st per patient per annum (1st PPPA) technique. Welsh data cover 2016–18. Column 3. National EASR of BCC and cSCC 2013–18, using all registered tumours (All registered) technique. Lower and upper 95% confidence intervals are indicated by the dotted lines

In England, the EASR of BCC using 1^st^ PPPA method in 2013–18 was 370.0 PY (95% CI 369.0, 371.1) in males and 231.1 PY (95% CI 230.3, 231.8) in females. The incidence rate of BCC increased by an average of 6.2 PY over the study period, although this was non‐significant (95% CI −0.1, 12.5) (Figure 3). The EASR of cSCC was 120.5 PY (95% CI 119.9, 121.1) in males and 46.2 PY (95% CI 45.9, 46.5) in females in the same period (Table 2). The incidence rate of cSCC increased by an average of 2.8 PY over the study period (95% CI 1.7, 4.0).

**TABLE 2:** KC EASRs in UK countries 2016‐18 using 1^st^ PPPA method with 95% confidence intervals. Scottish data based on registration of all cSCC tumours (all registered technique)

Country	Keratinocyte	Males	Females	Persons
England	BCC	381.0 (379.5, 382.4)	237.8 (236.8, 238.9)	309.4 (308.5, 310.3)
	cSCC	126.4 (125.5, 127.2)	48.2 (47.7, 48.6)	87.3 (86.8, 87.8)
Scotland	BCC	345.8 (341.4, 350.3)	204.3 (201.3, 207.4)	275.1 (272.4, 277.8)
	cSCC	114.6 (111.9, 117.4)	36.2 (35.0, 37.5)	75.4 (73.9, 76.9)
N. Ireland	BCC	384.6 (376.1, 393.1)	226.5 (220.8, 232.3)	294.7 (289.8, 299.6)
	cSCC	169.9 (163.9, 175.9)	59.2 (56.3, 62.1)	104.6 (101.6, 107.5)
Wales	BCC	420.9 (414.8, 427.1)	252.0 (247.6, 256.3)	336.4 (332.7, 340.2)
	cSCC	160.9 (157.0, 164.9)	61.0 (58.9, 63.1)	110.9 (108.7, 113.2)

Abbreviations: BCC, basal cell carcinoma; cSCC, cutaneous squamous cell carcinoma; EASRs, European Age‐Standardised Rates; KC, keratinocyte cancers; PPPA, per patient per annum.

In Scotland, the EASR of BCC 1^st^ PPPA in 2013–18 was 339.3 PY (95% CI 336.1, 342.5) in males and 201.2 PY (95% CI 199.1, 203.4) in females. The incidence rate of BCC increased by an average of 4.1 PY, although this was statistically non‐significant (95% CI −2.9, 11.0). The EASR of cSCC was 112.0 PY (95% CI 110.1, 114.0) in males and 36.5 PY (95% CI 35.6, 37.4) in females. The incidence rate of cSCC increased by an average of 1.4 PY over the study period (95% CI 0.6, 2.2). Scotland had the lowest rate of KC in the UK overall.

In Northern Ireland, the EASR of BCC in 2013–18 was 372.3 PY (95% CI 366.3, 378.3) in males and 219.3 PY (95% CI 215.3, 223.4) in females. Incidence rate of BCC increased by an average of 5.9 PY (95% CI 1.4, 10.5). The EASR of cSCC was 165.5 PY (95% CI 161.3, 169.8 PY) in males and 56.9 PY (95% CI 54.9, 59.0) in females. The incidence rate of cSCC increased by an average of 1.8 PY over the study period (95% CI 0.1, 3.5).

In Wales, the EASR of BCC in 2016–18 was 420.9 PY (95% CI 414.8, 427.1 PY) in males and 252.0 PY (95% CI 247.6, 256.3 PY) in females. The EASR of cSCC was 160.9 PY (95% CI 157.0, 164.9) in males and 61.0 PY (95% CI 58.9, 63.1) in females. Welsh data for the prior years was not available due to incomplete data collection.

### 4.4 Regional differences

The EASR of BCC from 2013 to 2018 was 10% lower in Scotland and 5% lower in Northern Ireland than the corresponding English rate (IRR = 0.90, 95% CI 0.90, 0.90 and IRR = 0.95, 95% CI 0.94, 0.95, respectively). The BCC incidence rate in Wales was higher than England’s in 2016 and increased to be 33.0 tumours PY higher than rate in England in 2018 and 9% higher than English BCC rate in 2016–2018 (IRR = 1.09, 95% CI 1.08, 1.09).

The EASR of cSCC was on average 21% higher in Northern Ireland than in England for all the years from 2013 to 2018 (IRR = 1.21, 95% CI 1.21, 1.22). The cSCC incidence rate in Wales was 6% higher than the Northern Irish rate from 2016 to 2018 (IRR = 1.06, 95% CI 1.05, 1.07), it increased from a similar level to that of Northern Ireland in 2016 to slightly higher by only 10.0 PY in 2018. EASR of cSCC in Wales was 27% higher than English rate 2016–2018 (IRR = 1.27, 95% CI 1.27, 1.28).

**FIGURE 4:**
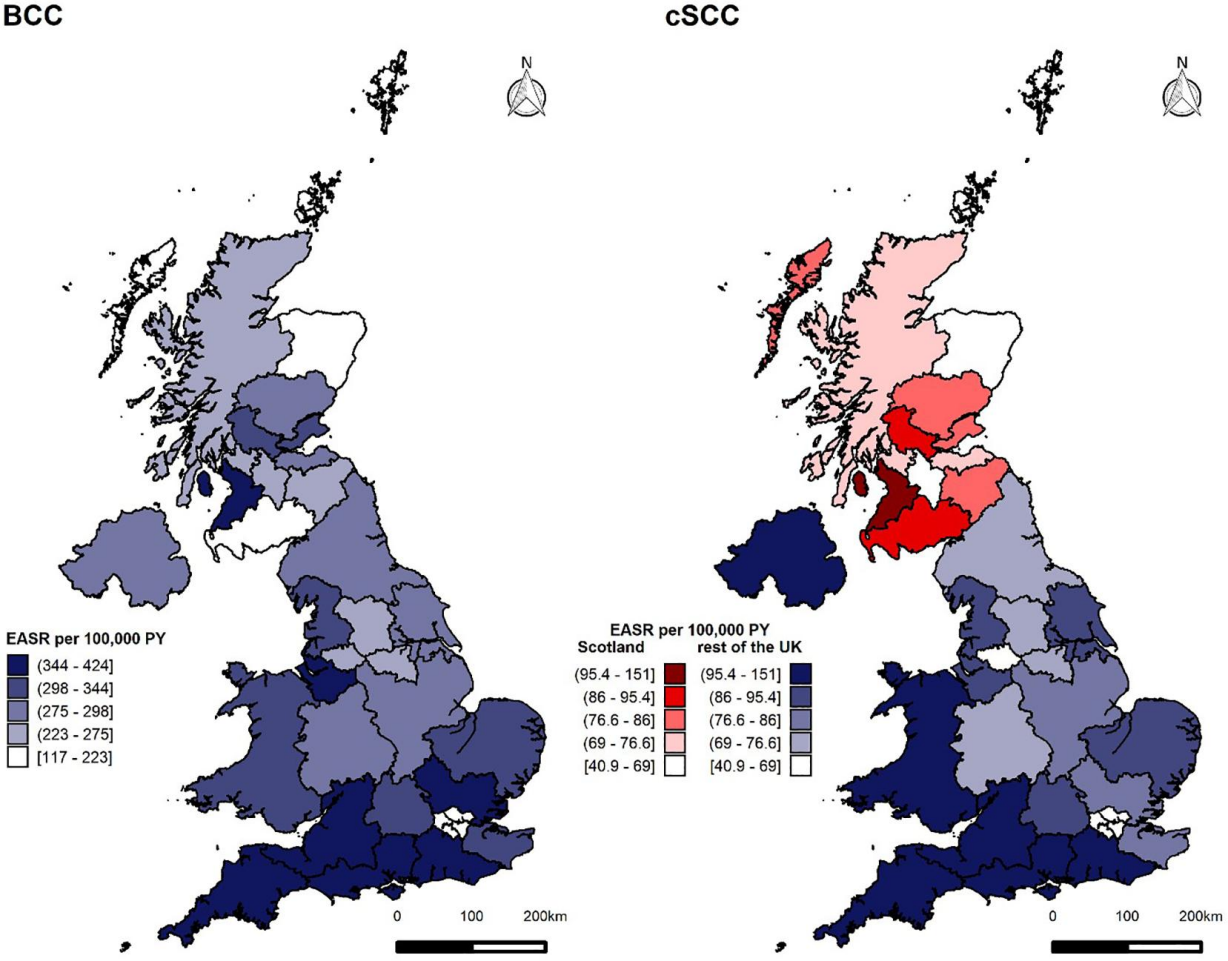
Regional variation in basal cell carcinoma (BCC) and cutaneous squamous cell carcinoma (cSCC) European Age‐Standardised Rate (EASR) incidence rate in England (Cancer Alliances), Scotland (NHS Boards), Wales and N. Ireland using first per patient per annum (1st PPPA) technique, except for Scotland where all cSCC are registered. Left. Regional EASR of BCCs 2016–18. Right. Regional EASR of cSCC 2016–18, Scottish data based on registration of all cSCC tumours (all registered technique)

The incidence rates of first KC per patient per annum differ among Cancer Alliances (England) and NHS Boards (Scotland) with the highest rates in southern and coastal regions in 2016–18 (Figure 4). The highest EASR of BCC was observed in Peninsula (424.1 PY [95% CI 418.8, 429.4]) and lowest in Dumfries and Galloway (117.3 PY [95% CI 108.2, 126.8] PY), although data extraction issues for Dumfries and Galloway have been highlighted by the Scottish Cancer Registry. The highest EASR of cSCC was in Peninsula (150.6 PY [95% CI 147.4, 153.8]) and lowest in Orkney (40.9 PY). The EASRs were generally lower in Scottish NHS Boards compared with the EASRs of English Cancer Alliances. EASR in Wales remained lower than regional rates in cancer alliances predominantly in Southern England including Peninsula, Surrey and Sussex, Wessex, East of England—South, Somerset, Wiltshire, Avon and Gloucestershire and Thames Valley, as well as Cheshire and Merseyside in the North of England as per Figure 4. EASR in Wales was higher than English cSCC rate in 2016–2018 but remained lower than regional rates in southern England including Peninsula and Surrey and Sussex.

### 4.5 Age‐specific rates in England

**FIGURE 5:**
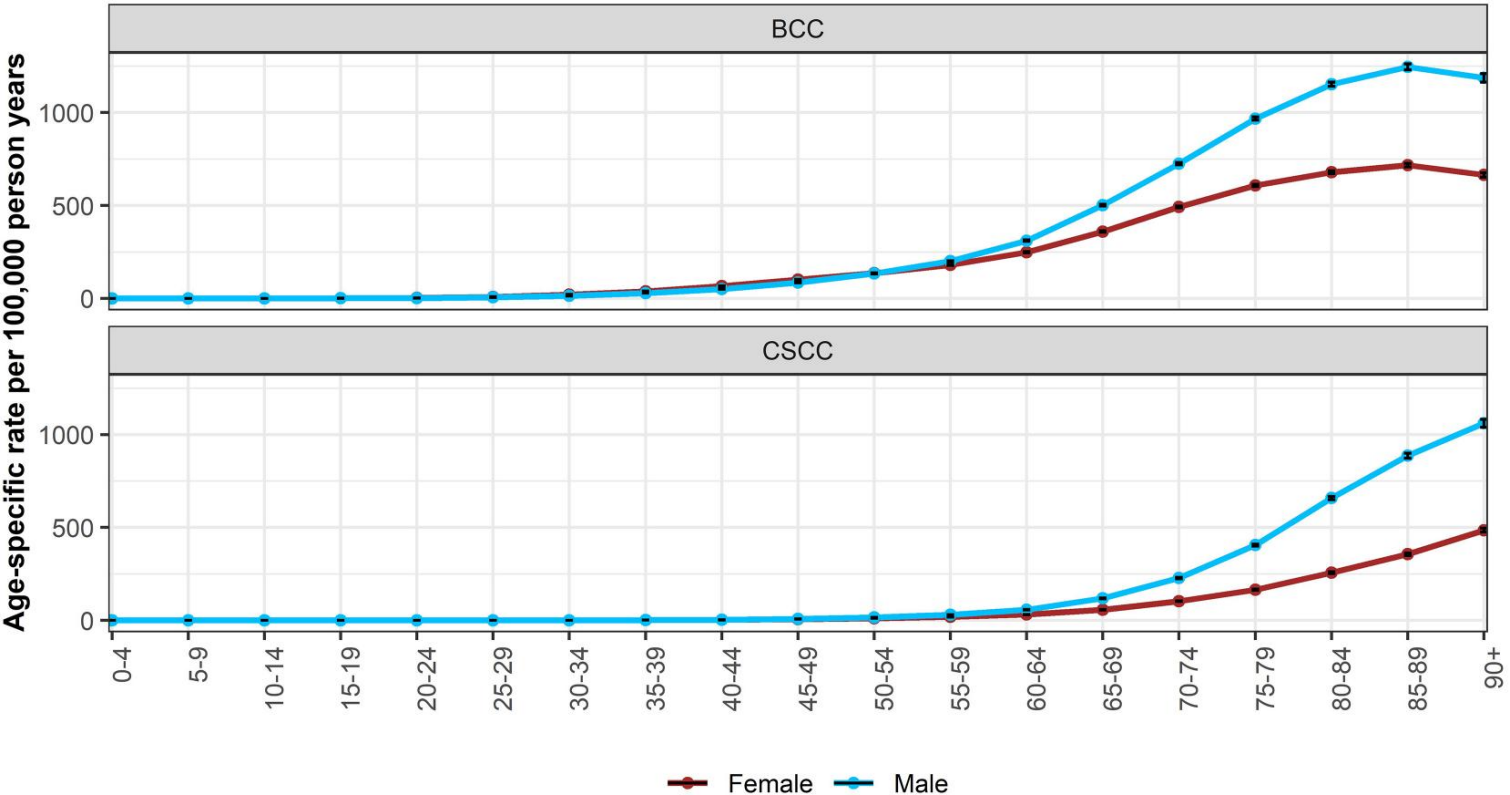
Age‐specific rates of basal cell carcinoma and cutaneous squamous cell carcinoma in males and females 2013–18, England. Using data for first tumour all‐time

The sex‐specific rates of BCC and cSCC are higher in males than females (IRR = 1.44, 95% CI 1.43, 1.45 and IRR = 2.34, 95% CI 2.32, 2.37, respectively). Interestingly, a decline in BCC incidence can be observed in patients above 90 years old (Figure 5). On average, the incidence of BCC was 5% and 7% lower in patients aged above 90 than 85–89‐year olds in males and females, respectively (IRR = 0.95, 95% CI 0.93, 0.98 and IRR = 0.93, 95% CI 0.91, 0.95).

**FIGURE 6:**
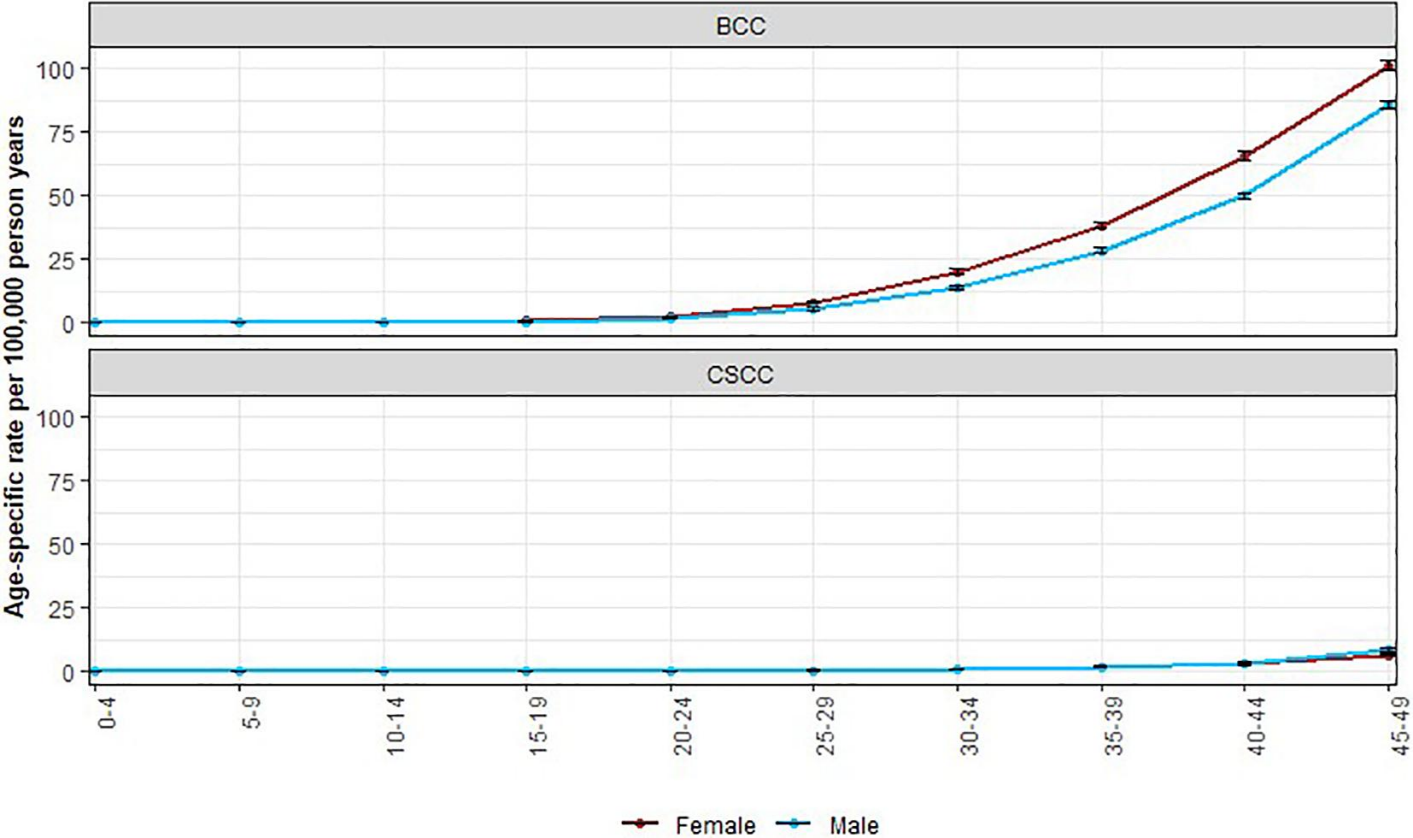
Age‐specific rates of basal cell carcinoma and cutaneous squamous cell carcinoma in males and females under 50 years old, 2013–18, England. Using data for first tumour all‐time

The sex‐specific incidence rate begins to increase from the age of 25–29 for BCC but remains low for cSCC. Incidence rate of BCC is lower in males than females under the age of 50 (IRR = 0.78, 95% CI 0.77, 0.80). On the contrary, the incidence of cSCC in patients under 50 is higher in males than in females (IRR = 1.25, 95% CI 1.16, 1.35) (Figure 6).

**FIGURE 7:**
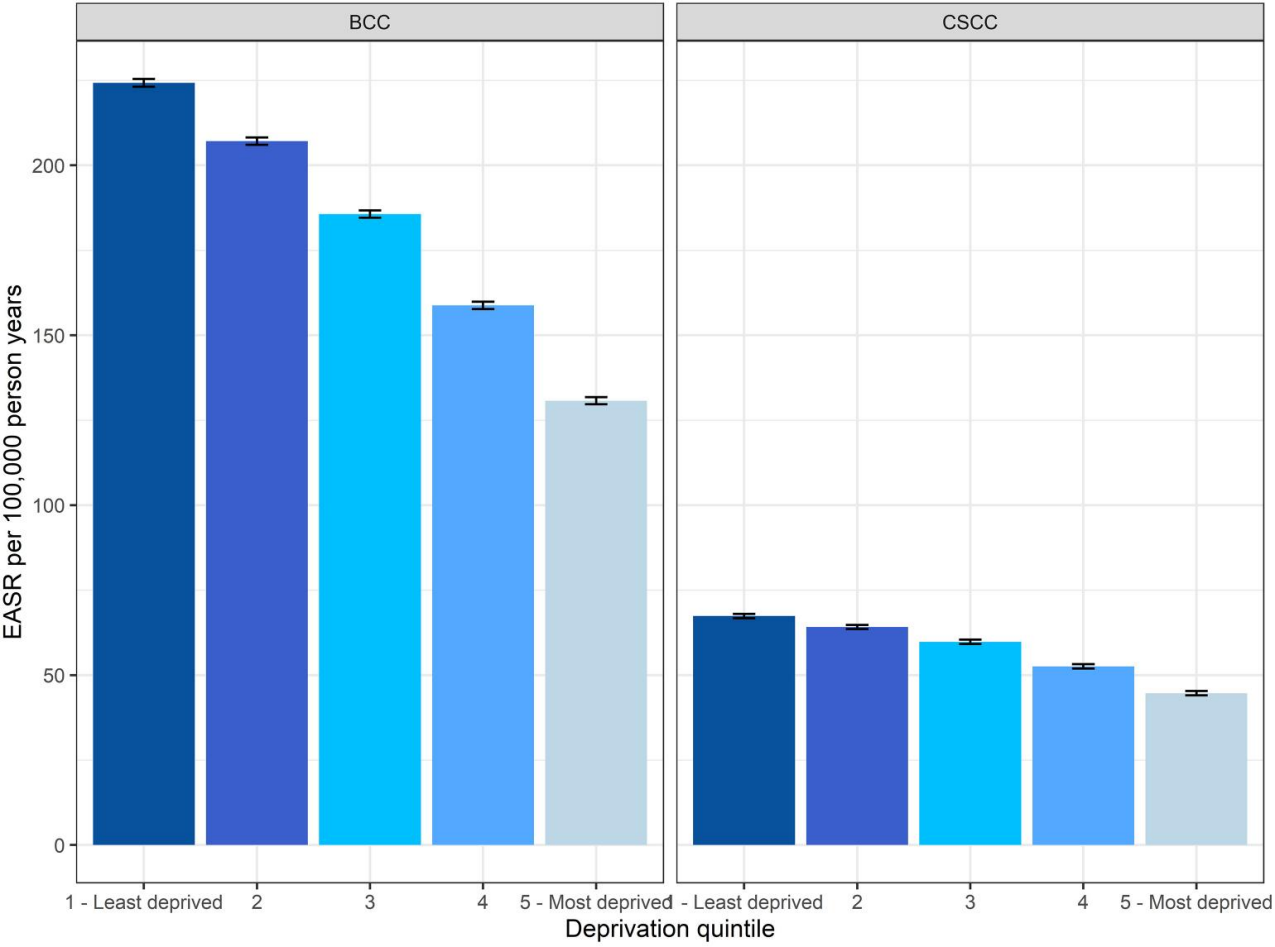
Age‐standardised incidence rates of basal cell carcinoma and cutaneous squamous cell carcinoma by deprivation level in 2013–2018, England. Using first tumour all‐time data. The error bars indicate 95% confidence intervals

The incidence of both BCC and cSCC shows an inverse relationship with local area deprivation level. On average, the incidence of BCC and cSCC was 179% and 155% higher in the least deprived areas compared to most deprived areas (IRR = 1.79, 95% CI 1.79, 1.80 and IRR = 1.55, 95% CI 1.53, 1.58, respectively) (Figure 7).

### 4.6 Lifetime incidence of NMSC

Approximately one in five (19.7%) people will develop at least one BCC, cSCC or other non‐melanoma skin cancer at some point in their lifetime in England, this equates to approximately one in four (22.3%) males and one in six females (17.5%).

### 4.7 Validation of the 1st PPPA method

Of 100 randomly selected patients with BCC in England reported in 2018 there were no incorrect diagnoses, however seven individuals' tumours were originally diagnosed in a previous calendar year which led to the same tumour being counted in both the previous calendar year and 2018 when using 1st PPPA method. There were 4 individuals with three BCCs and 13 individuals with two BCCs in the same calendar year. Therefore, the 1st PPPA method indicates an underestimation of approximately 14 BCC tumours per 100 patients compared to the actual number of new primary diagnoses a year, as estimated by this review of registry data by a trained dermatologist.

Of 100 randomly selected patients with cSCC reported in 2018, three had incorrect diagnoses (two Bowen’s disease, one BCC), and two individuals' tumours were originally diagnosed in a previous calendar year. There were seven individuals with two cSCC in the same calendar year. As a result, an underestimation of approximately two cSCC tumours per 100 patients was recorded, compared to the actual number of new primary diagnoses a year, as estimated by this review.

In England, the difference in tumour count between 1st PP and 1st PPPA methods was 59.2% for BCC and 37.2% for SCC.

## 5 DISCUSSION

We have, for the first time, estimated the incidence of KC across the whole of the UK, accounting for an average of 234 861 cases per year in the UK from 2016 to 2018 using 1st PPPA method (184 280 BCC and 50 582 cSCC).

Substantial increases in cSCC incidence in England, Scotland and Northern Ireland can be detected for the period of 2013 to 2018, and the incidence of cSCC also increased in Wales from 2016 to 2018. By contrast, however, the pattern of annual change in the incidence of BCC across the nations differs.

Using the 1st PPPA method, trends in BCC incidence increased in England and Scotland from 2013 to 2015, but more steeply in England. During the same period, the BCC incidence in Northern Ireland increased slightly. By 2018, the rate was very similar in England and Northern Ireland, and the rate in Scotland was lower than the English rate. In England, the incidence of BCC declined slightly from 2016 to 2018 and the overall trend across 2013‐18 is not statistically significant. In Scotland, the incidence of BCC shows some variability, declining in 2017 before increasing again in 2018, and the overall trend across 2013–18 was also not statistically significant. In Northern Ireland, the incidence of BCC increased significantly over the study period, and in Wales, the incidence of BCC increased from 2016 to 2018; however, we are limited to only 3 years of data in Wales. Although higher than the overall English BCC rate in 2016–2018, the EASR in Wales remained lower than regional rates in cancer alliances in Southern England including Peninsula, Surrey and Sussex, Wessex, East of England—South, Somerset, Wiltshire, Avon and Gloucestershire and Thames Valley as well as Cheshire and Merseyside in the North of England as per Figure 4.

Similarly, using 1st PPPA for cSCC incidence EASR, the rate was higher in Northern Ireland than in England for all the years from 2013 to 2018. The cSCC incidence rate in Wales was higher than the Northern Irish rate from 2016 to 2018. The EASR in Wales was higher than the overall English cSCC rate in 2016–2018 but remained lower than regional rates in southern England including Peninsula and Surrey and Sussex. The incidence of cSCC in Scotland cannot be directly compared with the other UK registry data owing to differences in practice. The Scottish registry has provided all registered cSCC instead of 1st PPPA cSCC as it is more accurate, however, this limits comparability with the other nations. The differences in counting methods must be considered when comparing data across UK nations. However, using the all‐registered method, the EASR incidence rate for Scotland is relatively stable from 2013 to 2018.

Cancer Alliances in London and in the Northern parts of Scotland had the lowest KC EASRs. The regional variation could be explained by the decreased Ultraviolet light radiation exposure in the higher latitudes, ethnicity and lifestyle factors such as indoor versus outdoor occupations. The cause for the differences across countries and regions may be multifactorial, there may be data collection or registration issues, or it may be the effect of ethnicity, behaviour or occupation differences. A similar ratio of BCC to cSCC is seen in some regions of England but further investigation is required to understand the underlying cause of the difference seen.

The KC incidence rates are higher among males in the more advanced age groups compared to females likely reflecting a lifetime of sun exposure as outdoor occupations were more common in males than females. Interestingly, the incidence of BCC, but not cSCC is higher in younger females than males. The higher levels in younger women could be a result of attitudes to sunbathing and sunbed use or greater health awareness in young women, who are more likely than young men to seek medical attention at an earlier stage and be more health aware.^10^ The decreasing incidence in BCC patients above the age of 90 is likely the result of the risk‐benefit consideration of referral or biopsy in elderly frail patients and is not seen in cSCC patients.^11^

The fall in BCC in 2017 and 2018 incidence of the first tumour per patient all‐time technique in England could be attributable to the improved cancer registration and collection from 2013 onwards resulting in an overinflated count of presumed first KCs if previous tumours had not been registered. However, this would not affect the first per patient per annum incidence which also shows a plateau effect. The 1^st^ PPPA data is generated from surgical specimens and therefore tumours treated conservatively or by photodynamic therapy, cryotherapy or topical chemotherapy will not be counted as they do not produce pathology reports which make up the majority of skin cancer registrations. The plateau effect might indicate a move towards these treatment modalities especially in the older population, where greater awareness of best practice when discussing treatment options with elderly patients as well as prolonged waiting lists may encourage conservative management of patients with BCC where appropriate.

The increase in cSCC as compared to BCC incidence could be due to increasing numbers of elderly and immunosuppressed patients who are at higher risk of developing an cSCC.^12^

The figures indicate a current lifetime risk of approximately one in five for NMSC in females and one in four for males. This estimate is much higher than the lifetime risk of melanoma (1 in 36 males and 1 in 47 females born after 1960 in the UK), which further highlights the burden of the disease and importance of early prevention strategy.^13^ However, the current probability method developed by CRUK based on a method by Sasieni et al uses a period approach, which assumes that current incidence rates will be constant through the individual’s lifetime. This means that the estimated theoretical risk of NMSC is based on NMSC incidence rates in 2013–18 only.

Overall, the high levels reported are likely an underestimation of the burden as validation of the 1st PPPA method indicates an underestimation of approximately 14 BCC tumours per 100 patients compared to the actual number of new primary diagnoses a year, as estimated by this review of registry data by a dermatologist.

Collaboration between UK national cancer registries is crucial to driving efforts to standardise KC incidence reporting. Therefore, our efforts are currently directed at the development of a gold standard for KC incidence reporting, that is, standardisation of morphology codes to identify KC and a universal implementation of the counting methods, that could be adopted by each country and aid future international comparisons including an annual UK‐wide KC incidence report.

Finally, to continue to improve our data collection at NCRAS we have developed a tool to compare pathology reports received at NCRAS in real‐time to local tumour counts to ensure complete KC data collection. This tool utilises unregistered data and therefore is not validated to report incidence but is intended to identify potential missing data. The tool can be accessed at https://cancerstats.ndrs.nhs.uk/COSD/Pathology/Keratinocyte. The data can be accessed through the secure Health and Social Care Network by all clinicians based in England eligible for an account on the CancerStats portal. We encourage all clinicians in England to compare their own KC data to that which we receive at NCRAS, for further information or if you need help accessing the data please contact zoe.venables@phe.gov.uk.

## 6 CONCLUSION

Accurate national incidence rates of BCC and cSCC are essential to healthcare planning. We highlight how common these tumours are by publishing the first ever lifetime incidence of NMSC. We report for the first time the incidence of KC in Wales, which further confirms the scale of the disease burden posed by these cancers on the UK nations. By providing a national report of KC incidence in 2013–2018, we aim to start the discussion around the incidence trends observed among countries that will lead to improved service planning and skin cancer prevention initiatives. Finally, we aim to continue to improve the data available to clinicians, researchers and the public to aid greater knowledge and understanding of skin cancers in the United Kingdom.

## CONFLICTS OF INTEREST

Dr George Millington is the current Academic Vice‐President of the BAD and Editor‐in‐Chief of Skin Health and Disease journal (SHD), Dr Tanya O. Bleiker is the President of the BAD and Prof. Mike Arden‐Jones is the chair of the BAD Research Subcommittee. Shehnaz Ahmed is employee of BAD.

## AUTHOR CONTRIBUTIONS

**M. Kwiatkowska:** Formal analysis; Writing – original draft; Writing – review & editing. **S. Ahmed:** Writing – review & editing. **M. Ardern‐Jones:** Writing – review & editing. **L. A. Bhatti:** Formal analysis; Writing – review & editing. **T. O. Bleiker:** Writing – review & editing. **A. Gavin:** Writing – review & editing. **S. Hussain:** Writing – review & editing. **D. W. Huws:** Writing – original draft; Writing – review & editing. **L. Irvine:** Supervision; Writing – review & editing. **S. M. Langan:** Methodology; Writing – review & editing. **H. Mitchell:** Data curation; Formal analysis. **R. Murphy:** Writing – review & editing. **C. M. Proby:** Writing – review & editing. **C. S. Thomson:** Writing – review & editing. **R. Thomas:** Formal analysis; Methodology. **C. Turner:** Methodology; Supervision; Writing – review & editing. **Z. C. Venables:** Conceptualization; Data curation; Formal analysis; Methodology; Supervision; Writing – original draft; Writing – review & editing.

## Supporting information

Supplementary Material

## Data Availability

The data that support the findings of this study are available in the supplementary material of this article.

## 1

N. Ireland and Wales:

KC were identified using topographical code C44 from the *International Statistical Classification of Diseases and Related Health Problems, Tenth Revision* (*ICD‐10*); and morphology codes from the *International Classification of Diseases for Oncology, Third Edition* (*ICD‐O‐2*):

BCC–ICD‐10 C44, ICD‐O‐3 8090, 8091, 8092, 8093, 8094, 8095, 8097.

cSCC–ICD‐10 C44, ICD‐O‐3 8050, 8051, 8052, 8054, 8070, 8071, 8072, 8073, 8074, 8075, 8076, 8077, 8078, 8082, 8083, 8084, 8085, 8086.

Other inclusion criteria applied to the cohort consist of standard methodology used at NCRAS, PHE.

Scotland:

KC were identified using topographical code C44 from the *International Statistical Classification of Diseases and Related Health Problems, Tenth Revision* (*ICD‐10*); and morphology codes from the *International Classification of Diseases for Oncology, Third Edition* (*ICD‐O‐3*):

BCC–ICD‐10 C44, ICD‐O‐3 8090‐8098.

cSCC–ICD‐10 C44, ICD‐O‐3 8050‐8078, 8083‐8084.
