# Correction to An updated report on the incidence and epidemiological trends of keratinocyte cancers in the United Kingdom 2013–2018

**DOI:** 10.1002/ski2.298

**Published:** 2023-10-07

In the article by Kwiatkowska et al.^1^ there is an error in the Discussion section.

The first sentence of the 9th paragraph of the Discussion section should read ‘The figures indicate a current lifetime risk of approximately one in six for NMSC in females and one in four for males.’

The authors apologize for this error.

## References

[1] Kwiatkowska M , Ahmed S , Ardern‐Jones M , Bhatti LA , Bleiker TO , Gavin A , et al. An updated report on the incidence and epidemiological trends of keratinocyte cancers in the United Kingdom 2013–2018. Skin Health Dis. 2022;1(4):e61–e63. 10.1002/ski2.61 PMC9060124 35663774

